# The Modification of Substrate in the Soilless Cultivation of Raspberries (*Rubus Idaeus* L.) as a Factor Stimulating the Biosynthesis of Selected Bioactive Compounds in Fruits

**DOI:** 10.3390/molecules28010118

**Published:** 2022-12-23

**Authors:** Maciej Balawejder, Natalia Matłok, Tomasz Piechowiak, Małgorzata Szostek, Ireneusz Kapusta, Marcin Niemiec, Monika Komorowska, Marek Wróbel, Krzysztof Mudryk, Anna Szeląg-Sikora, Pavel Neuberger, Maciej Kuboń

**Affiliations:** 1 Department of Chemistry and Food Toxicology, University of Rzeszow, St. Ćwiklińskiej 1a, 35-601 Rzeszow, Poland; 2 Department of Food and Agriculture Production Engineering, University of Rzeszow, St. Zelwerowicza 4, 35-601 Rzeszow, Poland; 3 Department of Soil Science, Environmental Chemistry and Hydrology, College of Natural Sciences, University of Rzeszow, Zelwerowicza 8b, 35-601 Rzeszow, Poland; 4 Department of Food Technology and Human Nutrition, University of Rzeszow, St. Zelwerowicza 4, 35-601 Rzeszow, Poland; 5 Department of Agricultural and Environmental Chemistry, Faculty of Agriculture and Economics, University of Agriculture in Krakow, Al. Mickiewicza 21, 31-120 Krakow, Poland; 6 Department of Mechanical Engineering and Agrophysics, Faculty of Production and Power Engineering, University of Agriculture in Krakow, 30-149 Krakow, Poland; 7 Department of Production Engineering, Logistics and Applied Computer Science, Faculty of Production and Power Engineering, University of Agriculture in Krakow, Balicka 116B, 30-149 Krakow, Poland; 8 Faculty of Engineering, Czech University of Life Sciences Prague, Kamycká 129, 165 21 Praha, Czech Republic

**Keywords:** *Rubus idaeus* L., soilless substrate, coconut fibre, sheep wool, polyphenols, cyanidin 3-*O*-sophoroside, cyanidin 3-*O*-glucosyl-rutinoside, vitamin C, antioxidant potential, enzymatic activity

## Abstract

Raspberry fruits are a valuable source of bioactive compounds. The study used the modification of the substrate (coconut fibre), consisting of the use of various organic and mineral additives, in the soilless cultivation of raspberries. The additives influenced the biosynthesis of bioactive compounds in the raspberry fruits by modifying the sorption properties and the abundance of the substrate. The influence of the additives on the content of polyphenols was determined as well as their profile (UPLC-MS), antioxidant potential (ABTS), vitamin C content, and the activity of selected enzymes that are markers of stress and resistance to abiotic factors. In the study, a significant effect of these additives was observed on the biosynthesis of polyphenols in raspberry fruit. The highest increase in the content of these compounds in relation to the control sample (substrate—100% coconut fibre), namely 37.7%, was recorded in the case of fruit produced on coconut substrate enriched with sheep wool. These fruits were also characterised by a significantly different profile of these compounds. These changes were caused by readily available ammonium nitrogen and free amino acids in the decomposition of proteins contained in the sheep wool. This was confirmed by the recorded content of chlorophyll SPAD in the plant leaves and the activity of selected enzymes, which proves a low level of stress and good condition of the plants.

## 1. Introduction

Raspberry (*Rubus idaeus* L.) has been cultivated in many regions of the world for several centuries. It has a number of varieties with different cultivation requirements and fruiting periods (summer and autumn raspberries) [1,2]. Depending on the variety, the produced fruits also differ in terms of the content of vitamins, bioactive compounds, and their shelf life. Most of the varieties are intended for field cultivation, and their fruits are suitable only for processing [3,4,5]. Dessert raspberries are grown mainly under cover in a soilless system using fertigation [6].

Various types of substrates are used for raspberry cultivation including peat substrate, perlite, coke, mineral wool, and coconut fibre. However, due to the susceptibility to recirculation, more and more natural substrates are being used including mainly coconut fibre. It is a cheaper alternative to mineral wool, also used in soilless cultivation [7,8,9,10]. Coconut fibre has an excellent air-to-water ratio, which makes it difficult to overwater in this type of cultivation. In addition, the fibre has a high cationic exchange, which means that unused minerals can be stored and used as needed at the right time. It is colonised by fungi of the genus *Trichoderma*, which have a protective function. Its mycelium covers the plant roots, thus protecting them against dangerous pathogens and stimulating root growth [11,12]. Coconut fibres are made of cellulose with limited sorption properties and increased susceptibility to degradation. These features require modification of coconut fibre substrates by adding various mineral and organic components that extend the life of these substrates and improve their physical properties [10,13].

Raspberry fruits are a very good source of dietary fibre, minerals (especially manganese), vitamin C, and bioactive ingredients, mainly polyphenols [14,15]. In addition, these fruits are characterised by strong antioxidant properties, which are useful for producing anti-inflammatory preparations [16,17]. The composition and content of bioactive compounds in raspberry fruits can be modified by fertilisation (using different nutrients for fertigation) or by changing the composition of the substrate [18].

The aim of the study was to modify the substrate in the soilless cultivation of raspberries (*Rubus idaeus* L.) to stimulate the biosynthesis of selected bioactive compounds in the fruit. The scope of the work included the preparation of substrates with various mineral and organic additives, which were tested in the greenhouse cultivation of raspberries. The fruit produced was analysed for the selected bioactive compounds (polyphenol content and profile, vitamin C content, antioxidant potential) and selected (enzymatic) stress markers were measured.

## 2. Results and Discussion

### 2.1. Bioactive Compounds

#### 2.1.1. Total Polyphenolic Content

Raspberry fruits are a valuable source of compounds with a documented health-promoting effect [19,20]. In a number of commercially available products, the main ingredient is raspberry isolate. One of the main groups of bioactive compounds in the components of raspberry isolates are polyphenols, which reduce the risk of metabolic diseases, especially cardiovascular, diabetes, obesity, and Alzheimer’s [21]. However, this effect is directly correlated with the content of these compounds in the raw material. It depends on the varietal characteristics and the substrate applied during cultivation and fertilisation [6,18]. Typical greenhouse cultivation of dessert raspberries is carried out in production pots, on a substrate made of coconut fibre [7]. This substrate is characterised by high sorption properties, but is devoid of the fertilising components that occur in soil cultivation. In the case of cultivation in coconut substrate, nutrients are supplied to plants by fertigation [22]. When using a single composition of the nutrient solution and the same method of application to plants (frequency and dose of the nutrient solution), the only possibility of modifying the conditions of plant growth and development is to change the composition of the substrate. This change can improve the biological properties of the produced raspberry fruit.

**Figure 1:**
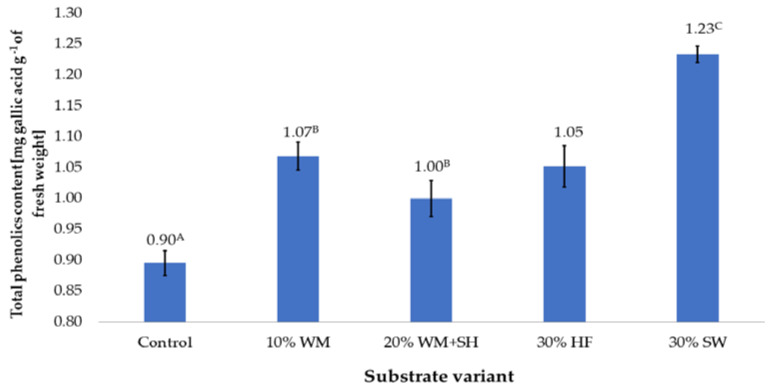
The total content of polyphenols in raspberry fruits, depending on the substrate used (n = 36). Note: Significant differences between the results, depending on the substrate variant used, are marked with different letters. Significant level at *p* < 0.05. Key: Control—100% coconut fibre; 10% WM—90% coconut fibre + 10% fragmented wood material; 20% WM + SH—80% coconut fibre + 10% fragmented wood material + 10% biochar from sunflower husk; 30% HF—70% coconut fibre + 30% hemp fibre; 30% SW—70% coconut fibre + 30% sheep wool.

The additives used for the coconut substrate were biochar (from sunflower husk) and organic components (an organic component obtained by steam treating fragmented wood, sheep wool, and hemp fibre). The addition of these ingredients significantly influenced the total content of polyphenols in the produced raspberry fruits (Figure 1).

Fruits produced on a coconut fibre substrate (control sample) had the lowest total content of polyphenols, which was 0.90 mg gallic acid per g^−1^ of fresh weight. Modification of the composition of the substrate, regardless of the additive, increased the content of polyphenols in the fruit. The highest increase in the content of these compounds, which was 37.7%, was recorded in the case of fruit produced on a coconut substrate enriched with sheep wool (30% SW).

A significant increase in the content of polyphenols was also observed in the case of the production of raspberry on a coconut substrate with organic additives (Figure 1). In the case of organic additives, the content of these components increased on average by 18.4% (addition of an organic component obtained by treating fragmented wood material with steam—10% WM). Due to its production method, it was assumed that this component would contain soluble components with a high degree of absorption, because they were obtained directly from wood tissue. Similar results were obtained in the case of raspberry production on a coconut substrate enriched with hemp fibre (30% HF). The total polyphenol content in these fruits was 17.5% higher than in the control sample. In the case of the 20% WM + SH substrate variant, a worse effect was observed with the addition of sunflower husk biochar. In this variant, the total content of polyphenols in fruits was 11.7% higher than in the control samples, but was significantly lower than in the other experiment variants (Figure 1).

A significant increase in polyphenol content was observed in raspberry fruit produced on a coconut substrate enriched with sheep wool (30% SW). The sheep wool, which is mainly composed of keratin protein, with a high nitrogen content of approximately 15% [23]. However, this nitrogen is not directly available to plants, but its supply is spread over time and depends on the degree and speed of protein decomposition, which is its source [24,25,26]. The high fertilisation efficiency of sheep wool protein was confirmed in the fertilisation of tomatoes (*Lycopersicon esculentum* L.) [22]. The authors showed increased plant growth under the influence of N released from protein, but did not investigate the content of bioactive compounds in the produced tomato fruits. On the other hand, in the case of the 20% WM + SH substrate variant, a worse effect was observed with the addition of sunflower husk biochar, which presumably limited the availability of nutrients contained in the organic component. Biochar has a high affinity for chemical compounds, which it binds through surface action, mainly adsorption [27]. This action can be so strong that it significantly limits the bioavailability of the ingredients contained in the organic component that is added to the substrate, but it can also affect the availability of nutrients applied to plants through fertigation.

#### 2.1.2. Profile of Polyphenolic Compounds

The profile of polyphenolic compounds identified by UPLC-PDA-MS/MS in raspberry fruits in relation to the substrate variant used is presented in Table 1.

**Figure 2:**
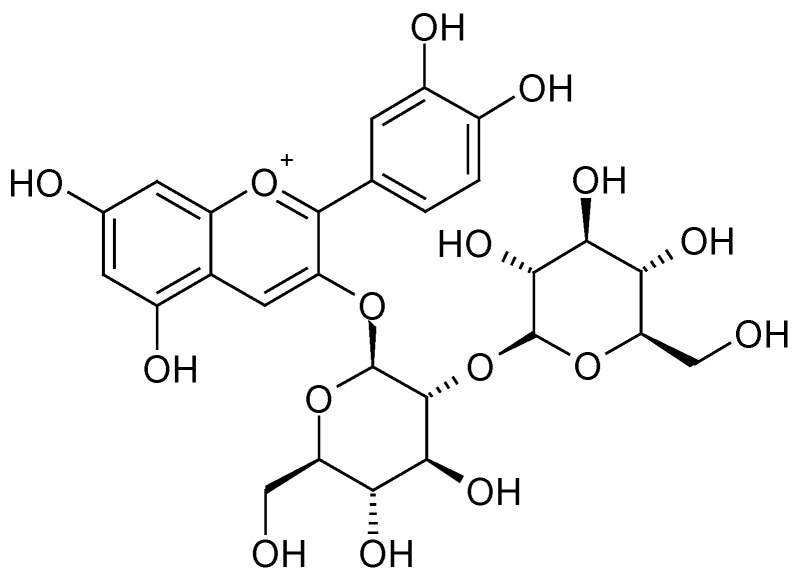
Cyanidin 3-*O*-sophoroside [28].

**Figure 3:**
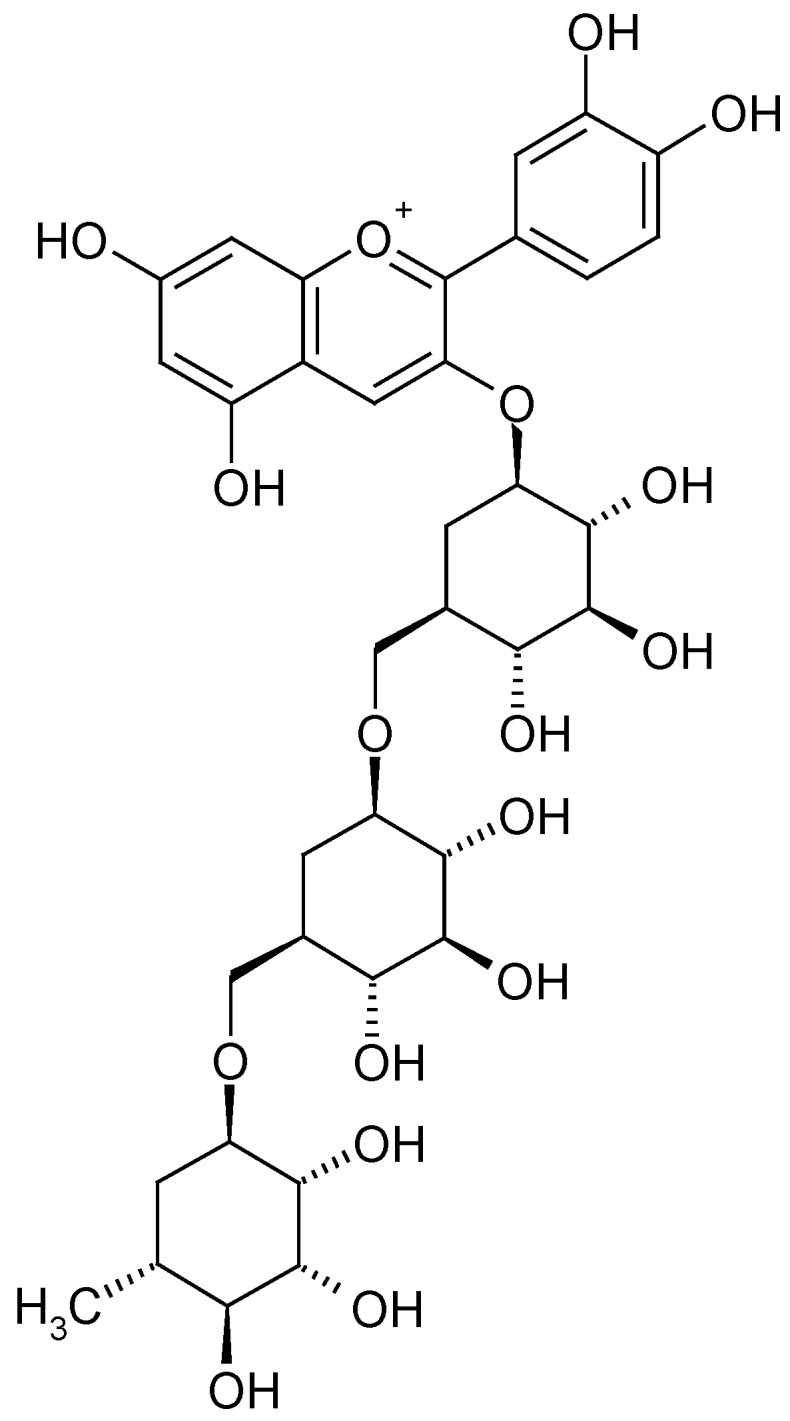
Cyanidin 3-*O*-glucosyl-rutinoside [29].

The highest content of polyphenols and changes in their profile were observed when using organic additives, especially those with fertilising properties. In raspberry fruits, regardless of the substrate used, the dominant ingredients were cyanidin 3-*O*-sophoroside (Figure 2) and cyanidin 3-*O*-glucosyl-rutinoside (Figure 3 and Table 1). In the case of the utilised substrates (exception 30% SW), the content of cyanidin 3-*O*-sophoroside was ~65%. On the other hand, in the case of fruit grown on a coconut substrate with the addition of sheep wool (30% SW), the proportion of this component in the mixture decreased by ~50%.

The studies by Balawejder et al. [25] and Olbrycht et al. [26] proved that under appropriate conditions, protein substances in the substrate are decomposed, releasing readily available ammonium nitrogen and free amino acids, which directly affect plant metabolism. Montesano et al. [30] showed that in the case of the nitrogen fertilisation of lettuce plants, the polyphenol profile changed depending on the dose of N. It was also shown that the antioxidant potential of lettuce leaves also changed under the influence of this fertiliser component. Please note that the additives applied to the coconut substrate did not change the quality of the profile of polyphenolic compounds (the same compounds were identified in all samples) (Table 1).

**Figure 4:**
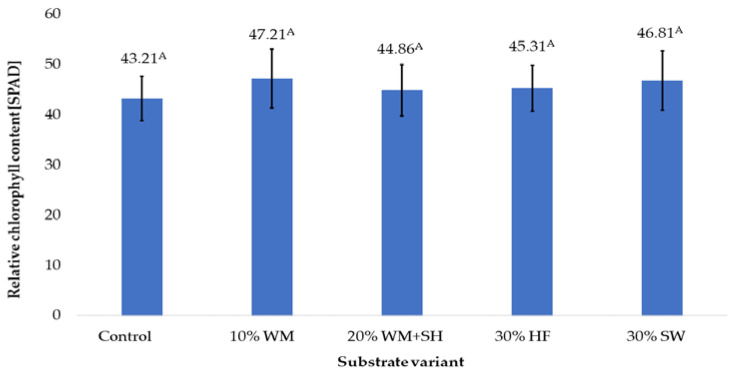
Relative chlorophyll content in raspberry fruits, depending on the substrate used (n = 36). Note: Significant differences between the results, depending on the substrate variant used, are marked with different letters. Significant level at *p* < 0.05. Key: Control—100% coconut fibre; 10% WM—90% coconut fibre + 10% fragmented wood material; 20% WM + SH—80% coconut fibre + 10% fragmented wood material + 10% biochar from sunflower husk; 30% HF—70% coconut fibre + 30% hemp fibre; 30% SW—70% coconut fibre + 30% sheep wool.

One of the parameters that determine the degree of nitrogen plant nutrition is the relative chlorophyll content of the leaf [25]. The measured soil plant analysis development (SPAD) values ranged from 43.21 (Control) to 47.21 (10% WM). It was also observed that the addition of sheep wool to the coconut substrate (30% SW) probably resulted in the release of available nitrogen, which directly led to a high level of chlorophyll content (SPAD 46.81). (Figure 4).

#### 2.1.3. Antioxidant Potential

The antioxidant potential is shaped by a number of low molecular weight substances and by selected enzymatic systems. One of the main components influencing this parameter is the content of polyphenolic compounds [31,32].

**Figure 5:**
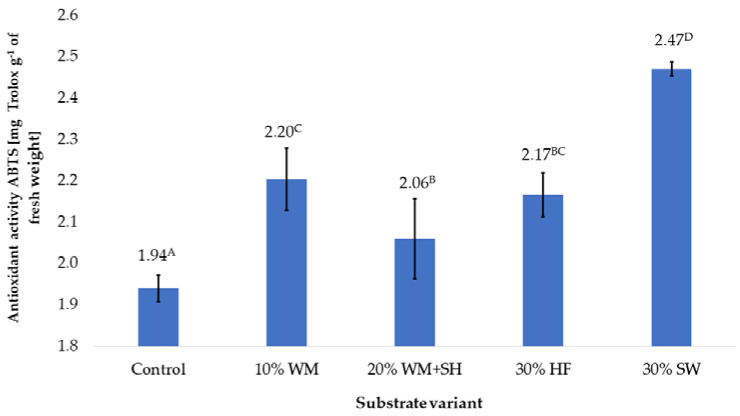
The antioxidant potential of raspberry fruit, depending on the substrate used (n = 36). Note: Significant differences between the results, depending on the substrate variant used, are marked with different letters. Significant level at *p* < 0.05. Key: Control—100% coconut fibre; 10% WM—90% coconut fibre + 10% fragmented wood material; 20% WM + SH—80% coconut fibre + 10% fragmented wood material + 10% biochar from sunflower husk; 30% HF—70% coconut fibre + 30% hemp fibre; 30% SW—70% coconut fibre + 30% sheep wool.

The additives applied to coconut fibre significantly influenced the antioxidant potential of the raspberry fruit (Figure 5). The results showed a similar effect as in the case of the polyphenol content on the antioxidant potential of the fruit produced. The highest antioxidant potential was found in raspberry fruit produced on a substrate with the addition of sheep wool (30% SW). Significant increases in the potential of fruit, compared to the control sample, were also recorded in the case of fruit produced on the 10% WM, 20% WM + SH, and 30% HF. Additives used on the substrates presumably act in the same way as the total content of polyphenols in raspberry fruit (Figure 1).

#### 2.1.4. Ascorbic Acid Content

Most products made from raspberry fruit contain significant amounts of vitamin C. This vitamin is an extremely important bioactive ingredient that contributes to the prevention of respiratory diseases, but its deficiency can cause other dysfunctions such as scurvy [33].

**Figure 6:**
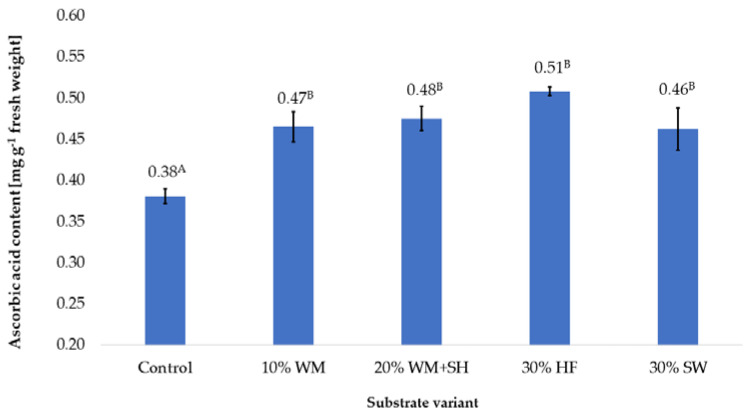
Vitamin C content in raspberry fruits, depending on the substrate used (n = 36). Note: Significant differences between the results, depending on the substrate variant used, are marked with different letters. Significant level at *p* < 0.05. Key: Control—100% coconut fibre; 10% WM—90% coconut fibre + 10% fragmented wood material; 20% WM + SH—80% coconut fibre + 10% fragmented wood material + 10% biochar from sunflower husk; 30% HF—70% coconut fibre + 30% hemp fibre; 30% SW—70% coconut fibre + 30% sheep wool.

In the case of the additives used on the coconut substrate, in each of the variants, a significant impact on the content of vitamin C in the produced raspberry fruits was produced (Figure 6). The highest content of this vitamin, 0.51 mg per g^−^^1^ of fresh weight, was recorded in the case of fruit produced on the substrate with the addition of hemp fibre (30% HF). This content was 31.3% higher than the content recorded in the control sample. The high vitamin C in the fruit produced on the substrate with this additive (30% HF) was most likely due to an additional source of stimulating factors. These factors can include bioactive substances in which Cannabis sativa L. plants and the dry substrate obtained from them abound [34].

In the case of the remaining variants of the substrate, similar vitamin C content was recorded, which were on average ~20% higher than the control sample. Presumably, the changes in retention and sorption properties caused by the additives used in the coconut substrate resulted in the modification of the vitamin C content in the produced raspberry fruits by changing the amount of nutrients available to plants from the applied nutrient solution. Similar effects of changing the efficiency of broccoli fertigation were observed by modifying the substrate with low-retention organic additives [35]. The authors found that the addition of humic substances adsorbed in the substrate increased the content of vitamin C in Broccoli oleracea L. by increasing the retention of nutrients.

### 2.2. Activity of Selected Enzymes

The activity of selected enzymes can be used as an indicator of the stress level of plants or as an indicator of the level of metabolic activity. Under conditions of stress caused by abiotic factors or increased metabolic activity in plant tissues, the level of reactive oxygen species (ROS), mainly hydrogen peroxide, increases. Attacks by pests, unfavourable weather conditions, or nutrient deficiencies, contribute to the increased production of hydrogen peroxide by peroxisomes. It should also be noted that as a result of increased photosynthesis, it is possible to convert water and molecular oxygen to hydrogen peroxide [36]. The presence of these molecules acts as a trigger that induces post-translational modifications that affect the functionality of target molecules such as proteins, peptides, or fatty acids [37]. This process can result in a change in the activity of enzymes such as catalase (CAT), superoxide dismutase (SOD), and glutathione peroxidase (GPOX). The activity of these enzymes also affects the level of antioxidants in the raw material, mainly the total content of polyphenols [38].

**Figure 7:**
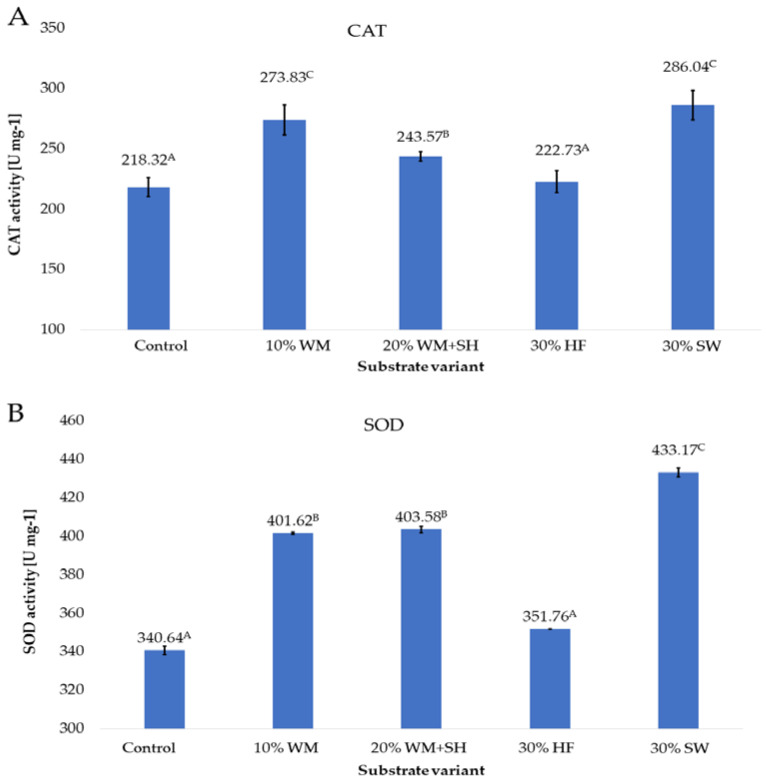

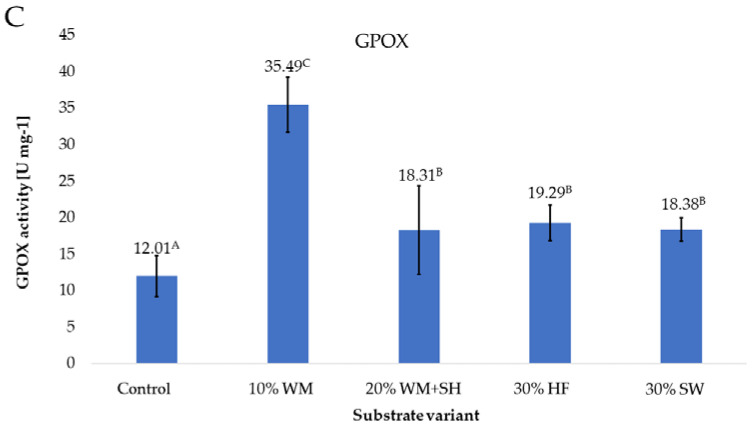
CAT (**A**), SOD (**B**), and GPOX (**C**) enzyme activity in raspberry fruits, depending on the substrate used (n = 36). Note: Significant differences between the results, depending on the substrate variant used, are marked with different letters. Significant level at *p* < 0.05. Key: Control—100% coconut fibre; 10% WM—90% coconut fibre + 10% fragmented wood material; 20% WM + SH—80% coconut fibre + 10% fragmented wood material + 10% biochar from sunflower husk; 30% HF—70% coconut fibre + 30% hemp fibre; 30% SW—70% coconut fibre + 30% sheep wool.

Applied to the substrate in the cultivation of raspberries, additive changes in the activity of the CAT, SOD, and GPOX enzymes (Figure 7 A–C) were observed.

The activity of the CAT enzyme in raspberry fruits produced on the substrate with the addition of sheep wool (30% SW) was 31.2% higher than the activity in the control sample (fruits produced on the coconut substrate). Due to the high activity of this enzyme, ROS is efficiently scavenged in the fruit. This directly affects the level of low molecular weight antioxidants, mainly polyphenols (Figure 1), and the total antioxidant potential of raspberry fruit (Figure 4). The good nutritional status of the plants, confirmed by the high relative chlorophyll content in the leaves (Figure 4), caused the activation of the plant’s defence mechanisms, which is indicated by the high activity of CAT (Figure 7A). A similarly high CAT activity recorded in raspberry fruits produced on coconut substrate with the addition of a concentrated organic component was obtained by treating fragmented wood material (10% WM) with steam. The level of activity of this enzyme was higher by 25.2% compared to the activity recorded in the control sample. The increase in this activity was most likely due to the presence of terpene substances including aromatic substances in the composition of the organic component that is an additive to this substrate, which, as Ninkuu et al. [39] demonstrated, influence the support of defence mechanisms in plants. A slightly lower activity of the CAT enzyme was recorded for fruits produced on the substrate with the addition of a concentrated organic component and biochar obtained from sunflower husk (20% WM + SH) and hemp fibre (30% HF). The results show that the profile of CAT activity is consistent with the profile of the total content of polyphenols and the total antioxidant activity of raspberry fruit, depending on the additive used in the substrate. This was confirmed by the observations of Matłok et al. [38], who showed a correlation between the activity of CAT and the content of low molecular weight antioxidants in the *Kalanchoe daigremontiana* plant material.

An important factor that generates hydrogen peroxide is the presence of a superoxide anion radical, which is formed by attaching an electron to an oxygen molecule in various types of biochemical reactions [40]. The SOD activity profile, which is similar to the CAT activity, indicates that the applied additives to the coconut fibre substrate similarly modulate the activity of the defence mechanisms of raspberry plants by increasing the degree of plant nutrition (Figure 7B) as a result of increasing the availability and supply of plant nutrients (S5) or through the presence of aromatic terpenes (10% WM and 20% WM + SH).

For many plants, the activity of the GPOX enzyme [41] can be a marker of the level of plant stress. The highest activity of this enzyme, 35.49 U mg^−1^ (Figure 7C), was recorded in the case of raspberry fruits produced on a substrate with the addition of a concentrated organic component (10% WM). Most likely, this is due to the presence in the composition of this component of organic substances with an aromatic structure, which are present in large amounts in the wood material. These substances are a stress factor for plants as a result of direct action [42], which directly affects the activity of the GPOX enzyme.

## 3. Materials and Methods

### 3.1. Production Experiment (Plant Growth Conditions)

A one-factor experiment was carried out with the greenhouse cultivation of dessert raspberries of the Malling™ Bella variety. Long-cane plants were planted in 11 litre production pots filled with substrate: control—100% coconut fibre; 10% WM—90% coconut fibre enriched with 10% concentrated organic component obtained by treating fragmented wood material with steam (a fibreboard by-product); 20% WM + SH—80% coconut fibre enriched with 10% concentrated organic component obtained by steam treating fragmented wood material (a fibreboard by-product) and 10% biochar obtained from sunflower husk; 30% HF—70% coconut fibre enriched with 30% hemp fibre; 30% SW—70% coconut fibre enriched with 30% sheep wool. The pot experiment was carried out in a completely randomised system, in three independent series. In particular series, each variant of the experiment was carried out in twelve repetitions. The plants were fertilised in a fertigation system with a nutrient substrate basis of the required EC conductivity, with the same dose of water in all variants.

### 3.2. Plant Material Preparation and Analysis

The method of preparing raspberry samples and determining the content of bioactive compounds, the polyphenolic compounds analysis, UPLC determination of polyphenolic compounds, enzymatic activity and relative chlorophyll content (SPAD), and statistical analysis methodology are described in detail in the attached Supplementary Materials (Enzymes extraction (S1); Activity of superoxide dismutase (S2); Activity of catalase (S3); Activity of guaiacol peroxidase (S4); determination of individual polyphenols from raspberry fruit (S5); sample preparation for antioxidant activity analysis (S6); total content of polyphenols (S7); antioxidant potential (ABTS) (S8); ascorbic acid content (S9)) [43,44,45].

### 3.3. Statistical Analysis

Multidimensional analysis of variance (ANOVA) of results was performed at the significance level α = 0.05 using STATISTICA 13.1 software (TIBCO Software, Inc. Hillview Avenue, Palo Alto, CA, USA). The mean values calculated from the three independent replications were statistically analysed by comparing the results between the variants of the experiment.

## 4. Conclusions

Raspberry fruits are a valuable source of bioactive compounds, mainly polyphenols and vitamin C. The study used the modification of substrates (coconut fibre) in soilless raspberry cultivation. Various organic additives were used: sheep wool, hemp fibres, a concentrated organic component obtained by steam treating fragmented wood material (a fibreboard by-product), and sunflower seed biochar. These additives influenced the biosynthesis of bioactive compounds in the studied raspberry fruits by modifying the sorption properties and the abundance of the substrate.

As a result, significant effect of these additives on the biosynthesis of polyphenols in raspberry fruit was observed. The highest increase in the content of these compounds was recorded in relation to the control sample (substrate—100% coconut fibre), which is 37.7%, was recorded in the case of fruit produced on a coconut substrate enriched with sheep wool. These fruits were also characterised by a significantly different profile of these compounds. This was caused by different activity of selected enzymes, which shows the low level of stress and good condition of plants. The remaining ingredients significantly modified the enzymatic activity of the fruits.

The greatest changes were observed after the application of a concentrated organic component obtained by steam treating fragmented wood material. It contained aromatic terpene derivatives with a proven impact on the stress level in plants. The other additives used in the substrate (i.e., sunflower husk biochar and hemp fibre) significantly stimulated the biosynthesis of bioactive compounds in the raspberry fruit. In the case of biochar, the stimulation mechanism was based on the physical modification of the sorption properties of the substrate, which resulted in an increase in the bioavailability of nutrients supplied to plants through fertigation. The addition of hemp fibre also resulted in a moderate stimulation of the biosynthesis of bioactive compounds in the fruit. This was due to the great similarity of the chemical properties of their fibres to coconut fibre, which is mainly made of cellulose. The biosynthesis of the bioactive compounds was most likely induced by the remaining components of the hemp plant. A similar relationship was observed for the other determined parameters such as total antioxidant potential and vitamin C content.

## Figures and Tables

**Table 1:** Individual phenolic compounds identified by UPLC-PDA-MS/MS in raspberry fruits depending on the substrate used.

No	Compound	RT	[M-H]^+/−^	Fragment Ions	Absorbance Maxima	Content [%]				
		(min)	(*m*/*z*)	(*m*/*z*)	(nm)	Control	10% WM	20% WM + SH	30% HF	30% SW
1	Caffeic acid glucoside	2.58	341^−^	179	299sh; 324	1.18	1.34	1.49	1.28	4.25
2	Cyanidin 3-*O*-sophoroside	2.77	611^+^	287	279; 509	64.58	66.09	65.56	65.99	36.26
3	Cyanidin 3-*O*-glucosyl-rutinoside	2.91	757^+^	611; 287	279; 517	16.79	14.28	13.31	13.83	13.06
4	Cyanidin 3-*O*-glucoside	3.04	449^+^	287	279; 515	4.33	6.81	7.30	7.40	9.24
5	Cyanidin 3-*O*-rutinoside	3.18	595^+^	287	278; 512	4.94	4.66	4.81	5.02	9.82
6	Procyanidin dimmer type B	3.23	577^−^	289	274	1.50	1.16	1.30	1.14	4.13
7	(+) Catechin	3.69	289^−^	144	274	1.74	1.62	1.71	1.51	4.21
8	Ellagic acid rhamnoside	3.97	447^−^	301	360	1.35	1.16	1.13	1.03	4.13
9	Casuarinin	4.11	935^−^	633; 301	244	0.87	0.70	0.75	0.68	3.61
10	Lambertianin C	4.20	1401^−^	633; 301	244	0.93	0.75	0.78	0.77	3.74
11	Ellagic acid pentoside	4.40	433^−^	301	360	0.86	0.65	0.86	0.66	3.74
12	Quercetin 3-*O*-rhamnoside	5.55	447^−^	301	255; 350	0.93	0.74	1.02	0.70	3.79

## Data Availability

Not applicable.

## Supplementary Materials

The following supporting information can be downloaded at: https://www.mdpi.com/article/10.3390/molecules28010118/s1, Enzyme extraction (S1) [46,47,48]; activity of superoxide dismutase (S2) [47]; activity of catalase (S3); activity of guaiacol peroxidase (S4) [48]; determination of individual polyphenols from raspberry fruit (S5) [49]; sample preparation for antioxidant activity analysis (S6) [50,51]; total phenolic content analysis (S7); antioxidant potential (ABTS) (S8); ascorbic acid content (S9).

## Sample Availability

Samples of the compounds are available from the authors as extracts collected during investigation.
