# Supplementary material for: The Modification of Substrate in the Soilless Cultivation of Raspberries (Rubus Idaeus L.) as a Factor Stimulating the Biosynthesis of Selected Bioactive Compounds in Fruits
Source: Molecules. 2022 Dec 23;28(1):118. doi: 10.3390/molecules28010118 (PMC9822297; doi:10.3390/molecules28010118)
Supplement: Supplementary file 1 [file molecules-28-00118-s001.zip › molecules-2054378-supplementary.pdf]

SUPPLEMENTARY MATERIALS

S1: Enzyme extraction

Briefly, 2 g of frozen (−67 °C) and milled fruit tissue was homogenised with 50 mg of polyvinylpolypyrrolidone and 4 mL of chilled 50 mM sodium phosphate buffer (pH 7.4) containing a protease inhibitor mixture and 0.05% Triton X-100. The homogenate was centrifuged at 10,000× *g* for 30 min (4 °C) and the supernatant was subjected to analysis [46–48].

S2: Activity of superoxide dismutase

SOD activity was assessed using a colorimetric method based on determining the degree of inhibition of epinephrine autoxidation by SOD present in the fruit extract. Briefly, 5 µL of the extract was mixed with 95 µL of 50 mM sodium carbonate buffer (pH 10.2) and 5 µL of 10 mM epinephrine. The kinetics of absorbance increase was measured for 5 min at 490 nm. One unit of SOD activity was defined as the amount of enzyme that inhibits adrenaline oxidation by 50% [47].

S3: Activity of catalase

CAT activity was assessed in accordance with the methodology presented by Hadwan and Ali (2018) with minor modification, which involved colorimetric estimation of H_2O_2 residue with ammonium metavanadate in the enzyme mixture comprising catalase from the plant extract. To 10 µL of enzyme extract, 80 µL 10 mM sodium phosphate buffer (pH, 7.4) and 20 µL of 10 mM H_2O_2 were added. After 10 min of incubation at 37 °C, 20 µL of 0.01 M ammonium metavanadate solution was added. The absorbance of the reaction mixture was measured at 425 nm after 10 min of incubation. One unit of CAT activity was defined as the amount of the enzyme, which results in the neutralisation of 1 mmol H_2O_2 during 1 min at 37 °C.

S4: Activity of guaiacol peroxidase

Briefly, 20 µL of enzyme extract was mixed with 80 µL of 50 mM citrate buffer (pH 5.0) and 20 µL of 8.26 mM guaiacol. The reaction was started by adding 20 µL of 8.8 mM H_2O_2. The absorbance changes were measured at 470 nm for 10 min. One unit of GPOX was defined as the amount of enzyme causing 0.01 changes in absorbance for 1 min [48].

S5: Determination of individual polyphenols from raspberry fruit

For the chromatographic investigation, the raspberry fruits were homogenised using a T 25 Ultra Turrax IKA homogeniser (Warsaw, Poland). Then, 20 g of homogenate was transferred to Falcon tubes (50 mL) and centrifuged at 7500 rpm for 10 min. (Eppendorf Centrifuge 5430; Hamburg, Germany). The supernatants before injection were diluted with the mobile phase 1:4 (*v*/*v*). The identification of individual polyphenolic compounds was carried out using the UPLC-PDA-MS method according to the procedure of Kapusta et al. (2017) [49].

S6: Sample preparation for antioxidant activity analysis

The fruit tissue (5 g) was homogenised with 20 mL of 75 % methanol (*v*/*v*). Next, the homogenate was shaken for 30 min (150 rpm) and centrifuged at 10.000*g* for 30 min. The supernatant was subjected for the ABTS antioxidant activity and total phenolic content assays [50,51].

S7: Total phenolic content analysis

The raspberry extract (5 µL) was mixed on the plate well with 95 µL of distilled water, 10 µL of Folin–Ciocalteu reagent, and 20 µL of 20% Na_2CO_3. After 30 min of incubation, the absorbance was

measured at 700 nm and the obtained results were expressed as gallic acid equivalent per 100 g of fruit tissue.

S8: Antioxidant activity against ABTS$^{\bullet+}$

Briefly, 2 µL of the raspberry extract was added to 148 µL ABTS radical solution. After 30 min of incubation in darkness, the absorbance was measured at 734 nm. The results were expressed as Trolox equivalent per 100 g of fresh fruit tissue.

S9: Ascorbic acid content

Vitamin C estimation was determined by the Folin–Ciocalteu reagent method by Porter [1]. Raspberry tissue (5 g) was homogenised with 15 mL of 1% trichloroacetic acid and centrifuged at 7500 g for 30 min. Next, to 50 µL of the supernatant, 25 µL of Folin–Ciocalteu reagent and 75 µL of 10% TCA were added. After incubation (30 min), the absorbance at λ = 760 nm was measured using a microplate reader. The results obtained were expressed as mg of vitamin C per 100 g of fruit tissue.
